# Complete Cocrystal Formation during Resonant Acoustic Wet Granulation: Effect of Granulation Liquids

**DOI:** 10.3390/pharmaceutics13010056

**Published:** 2021-01-04

**Authors:** Ryoma Tanaka, Supisara Osotprasit, Jomjai Peerapattana, Kazuhide Ashizawa, Yusuke Hattori, Makoto Otsuka

**Affiliations:** 1 Graduate School of Pharmaceutical Sciences, Musashino University, 1-1-20 Shin-machi, Nishi-Tokyo, Tokyo 202-8585, Japan; g1878005@stu.musashino-u.ac.jp (R.T.); yhattori@musashino-u.ac.jp (Y.H.); 2 Center for Research and Development of Herbal Health Products, Faculty of Pharmaceutical Sciences, Khon Kaen University, Khon Kaen 40002, Thailand; supisara.jane@gmail.com (S.O.); jomsuj@kku.ac.th (J.P.); 3 Research Institute of Pharmaceutical Sciences, Musashino University, 1-1-20 Shin-machi, Nishi-Tokyo, Tokyo 202-8585, Japan; k-ashizawa@beach.ocn.ne.jp

**Keywords:** cocrystal, crystallization, solvate, hydrate, polymorphism, resonant acoustic mixer, granulation, formulation, dehydration, physical characterization

## Abstract

The manufacturing of solid pharmaceutical dosage forms composed of cocrystals requires numerous processes during which there is risk of dissociation into parent molecules. Resonant acoustic wet granulation (RAG) was devised in an effort to complete theophylline–citric acid (THPCIT) cocrystal formation during the granulation process, thereby reducing the number of operations. In addition, the influence of granulation liquid was investigated. A mixture of anhydrous THP (drug), anhydrous CIT (coformer), and hydroxypropyl cellulose (granulating agent) was processed by RAG with water or ethanol as a granulation liquid. The purposes were to (i) form granules using RAG as a breakthrough method; (ii) accomplish the cocrystallization during the integrated unit operation; and (iii) characterize the final solid product (i.e., tablet). The RAG procedure achieved complete cocrystal formation (>99%) and adequately sized granules (d50: >250 μm). The granulation using water (GW) facilitated formation of cocrystal hydrate which were then transformed into anhydrous cocrystal after drying, while the granulation using ethanol (GE) resulted in the formation of anhydrous cocrystal before and after drying. The dissolution of the highly dense GW tablet, which was compressed from granules including fine powder due to the dehydration, was slower than that of the GE tablet.

## 1. Introduction

Representative crystalline complex forms of active pharmaceutical ingredients (APIs) include solvates (including hydrates), salts, and cocrystals [1]. These composites can flexibly enhance the properties of APIs without changing the chemical structure, and improve the physicochemical, mechanical, and biopharmaceutical performance [2,3,4,5]. Cocrystals are a newer crystalline complex of APIs that have garnered attention over the last several years; Aitipamula et al. define them as “… crystalline single phase materials composed of two or more different molecular and/or ionic compounds generally in a stoichiometric ratio which are neither solvates nor simple salts” [6,7]. From a physical chemistry and regulatory perspective, cocrystals can be viewed as a special case of solvates and hydrates, wherein the second component, the coformer, is not a solvent (including water), and is typically nonvolatile [8]. In general, supramolecular, hydrogen-bond drug–drug or drug–coformer networks are formed in cocrystals [9]. In recent years, various cocrystal research have been carried out and have provided an avenue for the development of new crystal forms of a given API.

In pharmaceutical pre-formulation/formulation research, numerous techniques are used to obtain a molecular complex. Cocrystals can be synthesized using slow evaporation, slurry, or mechanical grinding (either neat or liquid-assisted) as suitable methods at laboratory scale [10,11,12,13]. On the other hand, recent industrial methods have included spray-drying and hot-melt extrusion [14,15]. After synthesizing the cocrystals, the problem arises that the cocrystals must be transitioned into a solid dosage form (i.e., granules or tablets) via several unit operations including milling, blending, granulation, and compression. In a case study of 1:1 caffeine22 glutaric acid cocrystal, ball milling induced polymorphic transformation from the stable form II to the metastable form I [16]. In other studies of 1:1 theophylline–glutaric acid and 1:1 caffeine–glutaric acid cocrystals, low-frequency Raman spectra and powder X-ray diffraction were used to monitor the dissociation of cocrystals and conversion to theophylline or caffeine monohydrate [17]. The case of scalable spray-drying and hot-melt extrusion for a cocrystal formation approach sometimes resulted in a low cocrystal yield due to partial amorphization [15,18]. These results are not surprising, because various pharmaceutical manufacturing processes significantly influence the final crystalline form of the product [19,20]. The process-induced stresses, such as mechanochemical effects, granulation liquid, momentary drying, and high temperature, are reported to accelerate the phase transitions [21].

One reported disadvantage is that the cocrystals can be dissociated or amorphized during the solid dosage manufacturing. Based on this background, we have considered the problem from a different angle and proposed a methodology of simultaneous cocrystallization and granulation during high-shear wet granulation (HSWG) [22,23]. This methodology obviates the need to synthesize the cocrystal prior to the manufacture of the dosage form, and thus contributes to the required reduction in the number of operations and continuous manufacturing [24]. However, as a limitation of our previous work, the complete cocrystal formation was difficult [22]. The HSWG should avoid adding a large volume of the granulation liquid, which facilitates the dissolution and reaction between drug and coformer (cocrystal components), in order to prevent overgranulation [25]. We have also shown that an excipient can be used to increase the reactivity of cocrystal components during the HSWG, but in that case a higher concentration of polymer is required [23]. As another idea to improve the problems presented by the use of this method even for simple compositions, it would be optimal to generate sufficient reaction movement between cocrystal components. The molecular movement of components during the process was known as a crucial factor to facilitate the cocrystal formation in the mechanistic insight [13].

In this study, in order to facilitate the cocrystal formation, our research group devised a resonant acoustic wet granulation (RAG) system. RAG is developed granulation system using a resonant acoustic mixer, which can achieve strong and rapid blending of materials by controlling the frequency and acceleration [26]. This mixer has been used effectively in the pharmaceutical industry for applications such as uniform and non-destructive blending, cocrystal formation, and dry coating [27,28,29]. Moreover, it is highlighted that the cocrystal formation by RAG are applicable to in situ monitoring and scale-up because these production systems using same resonant acoustic mixer have been reported [30,31]. We hypothesized that the resonant acoustic mixer would be able to blend a combination of materials energetically to induce the simultaneous cocrystal and granule formations even if a nonreactive drug and coformer were used. As a study compound, we selected theophylline–citric acid (THPCIT) cocrystal, because in pre-experiments we found that complete formation of the THPCIT cocrystal was impossible by the previous HSWG process. This work also assessed the influence of granulation liquid on the cocrystal products. For this assessment, we processed a mixture of anhydrous THP, anhydrous CIT, and hydroxypropyl cellulose (granulating agent; binder) by RAG with water (GW) or ethanol (GE) as a granulation liquid. Our purposes in this work were to (i) form granules of adequate size using RAG, (ii) accomplish the complete cocrystallization during the integrated unit operation, and (iii) characterize the final product.

## 2. Materials and Methods

### 2.1. Materials

**Figure 1:**
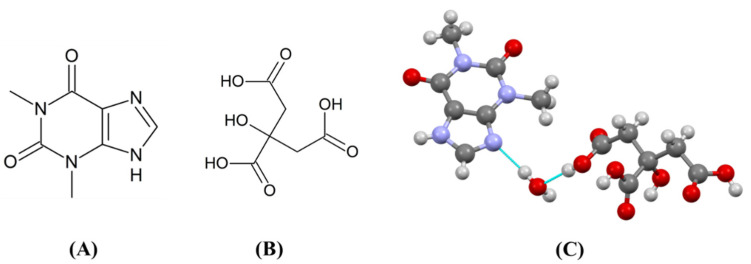
Chemical structures of (**A**) theophylline (THP), (**B**) citric acid (CIT), (**C**) THPCIT cocrystal hydrate.

Crystalline anhydrous theophylline (THP; drug; Figure 1A) and anhydrous citric acid (CIT; coformer; Figure 1B), were purchased from Shizuoka Caffeine (Shizuoka, Japan) and Fujifilm Wako Pure Chemical (Osaka, Japan), respectively. As a general granulation agent, hydroxypropyl cellulose (HPC-SSL; binder) was kindly gifted by NISSO (Tokyo, Japan). For the sake of comparison, the THP monohydrate was synthesized by vapor-mediated phase conversion [32]. The CIT monohydrate was purchased from Fujifilm Wako Pure Chemical. The 1:1 THPCIT cocrystal hydrate (Figure 1C; refcode KIGKAN in the Cambridge Structural Database) and anhydrous 1:1 THPCIT cocrystal were obtained by liquid-assisted grinding using 0.5 mL water and ethanol, respectively [33].

### 2.2. Resonant Acoustic Wet Granulation (RAG)

An equimolar mixture of anhydrous THP (9.4 g), anhydrous CIT (10 g), and HPC (0.6 g), consisting of a total of 20.0 g powder, was pre-mixed in a resonant acoustic mixer (LabRAM; Resodyn, MT) for 3 min, and then the wet granulation was carried out for 57 min with 4 mL water (20%; GW) or 5 mL ethanol (25%; GE). This RAG process (total of 60 min) facilitated cocrystal formation. The LabRAM condition was set with reference to the previous literature; the frequency and acceleration were 60 Hz and 980 m/s^2^, respectively [26]. The volume difference between liquids was decided with consideration for the volatilization amount of ethanol. All the wet samples were wet sieved through a 35-mesh (500 μm) screen and dried at 60 °C for 24 hr. The dried granules were sieved using a 100-mesh (150 μm) screen and collected.

High-shear wet granulation (HSWG) was also attempted using the same materials for comparison with RAG. For the HSWG, the same 20.0 g powder mixture was granulated with the same granulation liquids by a small-scale granulator (200 mL) [22]. The granulation was performed by kneading with each granulation liquid for 10 min and mashing for 20 min, and the process was repeated five times at 30 °C with 500 rpm agitator rotational speed to facilitate the cocrystallization [23]. After the granulation, wet sample granules were dried using the same condition.

### 2.3. X-ray Diffractometry (XRD)

The XRD patterns were collected by a RINT-Ultima III (Rigaku, Tokyo, Japan) at room temperature using Cu Kα radiation (40 kV × 40 mA). The angular range was 5–35° 2θ with a step size of 0.02° and the scanning was performed at 2°/min.

### 2.4. Differential Scanning Calorimetry (DSC)

The DSC profiles were obtained using a DSC7000X (Hitachi, Tokyo, Japan). Samples of approximately 5 mg were placed in an aluminum DSC pan, and then heated from 20 to 300 °C at a rate of 5 °C/min. The measurements were made under a dry nitrogen purge at 50 mL/min.

### 2.5. Scanning Electron Microscopy (SEM)

SEM images of anhydrous THP, the physical mixture (THP, CIT, and HPC bulk powder), and granules were taken by a JSM-6510LV (JEOL, Tokyo, Japan) for the morphological analysis. Samples were sprinkled on aluminum stubs with carbon tape and imaged. The acceleration voltage, magnification, and working distance were 1.0 kV, ×150, and 15 mm, respectively.

### 2.6. Flowability Analysis

Each sample was added to a graduated cylinder (50 mL) of approximately 10 mm in height of samples and the cylinder was tapped 100 times. The tapped samples were collected and weighted at the end of the experiments, and then the apparent density ($\rho_{B}$) and tapped density ($\rho_{T}$) were calculated. The standard indicators, i.e., the value of the Hausner ratio (HR) and Carr index (CI), were calculated using the following equations:

(1) $$HR=\frac{\rho_{T}}{\rho_{B}}$$

(2) $$CI\;\left(\%\right)=100\left(1-\frac{\rho_{B}}{\rho_{T}}\right)$$

In addition, the angle of repose (AR) was also measured using a tilting method. Approximately 30 mg of sample was fed into a holder and the holder was slowly tilted until the sample began to slide. AR was measured by reading the tilted angle.

### 2.7. Particle Size Distribution (PSD)

The volumetric PSDs were determined at a dry state using an LA-960V2 Dry laser diffraction/scattering analyzer (HORIBA, Kyoto, Japan). Approximately 5 g of each sample was added to the measurement chamber. The refractive index was set to 1.6 for all the measurements. Mie scattering theory was used for the analysis.

### 2.8. Tablet Compression and Characterization

Each of the 200 mg tablets was compressed using a Handtab-100 (Ichihashi Seiki, Kyoto, Japan) for tablet, with 8 mm diameter flat-faced punches. The compressing force and dwell time in tablet compression were 60 MPa and 5 s, respectively. The hardness on a PC-30 tablet hardness scale (Okada Seiko, Tokyo, Japan) was recorded.

Each tablet was scanned by a microfocus X-ray CT system, inspeXio SMX-100CT (Shimadzu, Kyoto, Japan), under a 90 kV × 110 μA condition. The scanning view number was 600, and images was acquired by 3 scans at 16 μm resolution. The image size was 8.265 × 8.265 × 8.265 mm^3^ (512 × 512 × 512 voxels: xyz dimension). The high absorption area was mapped using the VGStudioMAX 3.0 software package (Volume Graphics, Heidelberg, Germany). The mapping range was 38,300–40,000 gray value.

Dissolution testing of the tablet was performed in 900 mL water medium (37.5 ± 0.5 °C) using a DT-610 apparatus and V-530 spectrometer (JASCO, Tokyo, Japan). The paddle speed was 50 rpm and non-sink conditions were employed. Aliquots at the desired timepoints were automatically removed and the THP concentration was instantly measured using a spectrometer at 285 nm. Furthermore, the disintegration time of each tablet was measured during the dissolution testing.

## 3. Results and Discussion

### 3.1. Baseline Characterization

**Figure 2:**
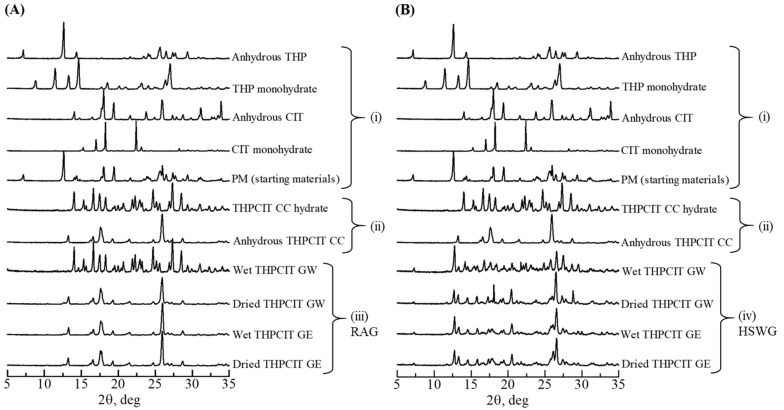
Overlay of powder XRD patterns of materials and experimental samples: (i) anhydrous and hydrate forms of THP and CIT, physical mixture of anhydrous THP and CIT (PM; starting materials); (ii) reference THPCIT cocrystals (CC; objective compounds); (**A**) (iii) wet and dried granules prepared by resonant acoustic granulation (RAG); and (**B**) (iv) the granules prepared by high-shear wet granulation (HSWG). Granulation was performed using water (GW) or ethanol (GE).

The XRD patterns of all components are shown in Figure 2. In addition, crystalline THP monohydrate was experimentally produced by vapor-mediated phase conversion in order to assess and compare the results [32]. For the same reason, anhydrous THP and anhydrous CIT (1:1 molar ratio) were grinded using 0.5 mL water and ethanol to prepare the THPCIT cocrystal hydrate and anhydrous THPCIT cocrystal, respectively [33]. The crystal structure of THP monohydrate and THPCIT cocrystal hydrate were already published in the Cambridge Structural Database (refcode: THEOPH01 and KIGKAN) [33,34]. Anhydrous cocrystal data were also available in a previous study [33]. All of these experimental XRD patterns were in agreement with the reported pattern (Figure 2).

### 3.2. Formation of Theophylline-Citric Acid (THPCIT) Cocrystal

One of the goals of the current study was to achieve simultaneous completion of THPCIT cocrystallization and granulation during the resonant wet acoustic granulation (RAG) system, as described in the Introduction. This completion was one of the challenges in our previous work [22,23]. Our project team hypothesized that the RAG system would accomplish a powerful mixing of materials that would easily induce cocrystal and granule formations. In order to reduce the complexity of the analysis, we used a simple composition consisting of only the drug, coformer, and binder. The 3% hydroxypropyl cellulose (HPC-SSL) is selected as a general binding agent in many granulations [35].

The role of granulation liquid was explored by granulation with water (GW) and ethanol (GE). After the RAG proceedings, the XRD pattern of wet GW granules was in excellent agreement with the pattern for the reference THPCIT cocrystal hydrate, with characteristic peaks at 14.1, 16.7, 17.4, 20.7, and 28.5° 2θ (Figure 2A). After the drying process, the cocrystal hydrate was converted into anhydrous cocrystal, which has peaks at 13.2, 16.2, 16.5, 17.6, 21.4, and 25.9° 2θ. In the case of the GE granules, the pattern of the wet GE granules agreed with reference anhydrous cocrystal, and it was not changed even after the drying process (Figure 2A). The completion of the cocrystal formation was discussed in detail later.

However, unlike when using RAG, the cocrystal formation was incomplete when the previous method of high-shear wet granulation (HSWG) was performed. Figure 2B shows the results of the HSWG. Wet THPCIT GW in the HSWG granule partially formed cocrystal hydrate, but also retained the peaks of the starting materials [anhydrous THP (7.2, 12.7, and 27.7° 2θ) and anhydrous CIT (18.0, 23.7, 31.3, and 33.9° 2θ)] and the hydrates [monohydrate of THP (14.6, 18.5, and 20.1° 2θ) and monohydrate CIT (16.9 and 22.4° 2θ)]. The peaks of hydrates were transformed into the peaks of the anhydrous after drying. The similarly incomplete cocrystallization was observed in the THPCIT GE granule using HSWG (Figure 2B). Hence, the partial reaction between THP and CIT and low cocrystal yield were proved in HSWG.

**Figure 3:**
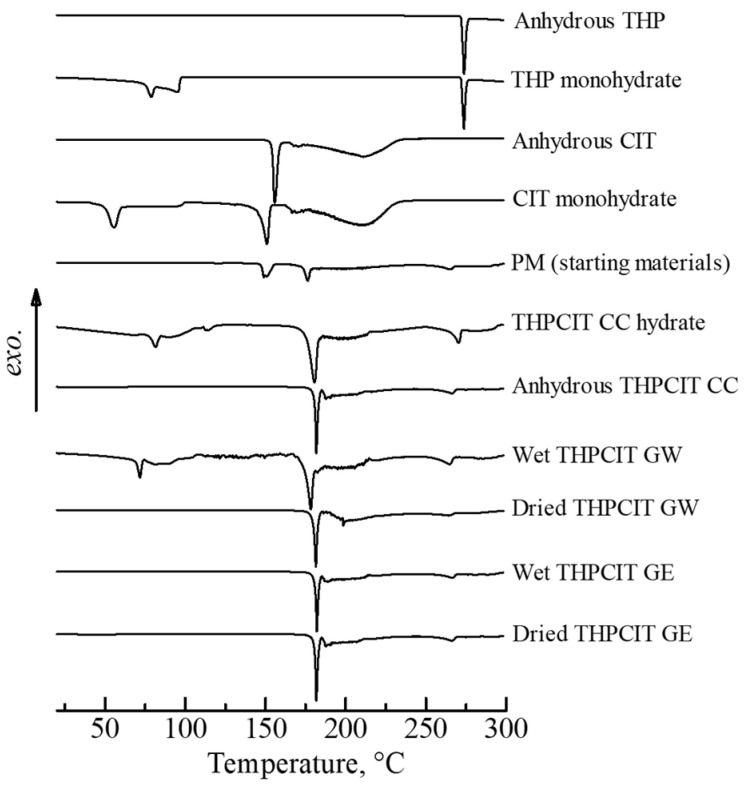
DSC heating curves of: anhydrous and hydrate forms of THP and CIT, physical mixture of anhydrous THP and CIT (PM; starting materials), reference THPCIT cocrystals (CC; objective compounds), and wet and dried granules prepared by resonant acoustic granulation (RAG) using water (GW) or ethanol (GE).

In order to clearly confirm the complete cocrystal formation via RAG process, the DSC profile was measured. DSC is known to be a more effective and sensitive approach for the detection of polymorphism and cocrystal formation than XRD [36,37]. In Figure 3, the thermal behavior of the reference THPCIT cocrystal hydrate showed an endothermic peak at 81.7 °C, which was immediately followed by an exothermic peak at 110.0 °C, suggesting the dehydration of crystal water and formation of anhydrous cocrystal, respectively. The second endothermic and exothermic were detected at 180.0 and 214.7 °C, which are the melting point of the anhydrous cocrystal and the crystallization temperature of stable THP theophylline. The third endothermic peak in the cocrystal hydrate at 270.1 °C was the melting point of the anhydrous THP. In addition, in the case of reference anhydrous THPCIT cocrystal, the finding that nearly the same endothermic and exothermic peaks were observed at 181.8, 213.7 and 268.5 °C was also attributed to the melting point of the anhydrous cocrystal, crystallization point of anhydrous THP formation, and melting point of the anhydrous THP, respectively. The DSC results in Figure 3 indicate that wet THPCIT GW from RAM formed pure cocrystal hydrates without any other components, because both of the hearting curves were in excellent agreement. The dried GW, wet GE, and dried GE from RAM had similar peaks of anhydrous cocrystal, suggesting they formed pure anhydrous cocrystal.

To summarize the results of XRD and DSC, the complete cocrystal formations were obtained through the RAG process with either water or ethanol. As mentioned earlier, our hypothesis was that the RAG can facilitate the cocrystal formation using the powerful and rapid mixing by resonant acoustic mixer. Recently, we documented fast homogeneous blending of the several pharmaceutical materials using resonant acoustic mixer, even in the case of low-dose APIs [26,27]. This blending was achieved by strong vibration controlling the frequency and acceleration. In this experiment, the high-speed blending system facilitated molecular movement between THA, CIT, and granulation liquid, which result in the cocrystal formation. This proposed RAG method was verified using the complete formation of ibuprofen–nicotinamide cocrystal and indomethacin–saccharin cocrystal.

### 3.3. Granule Morphology and Physical Properties

**Figure 4:**
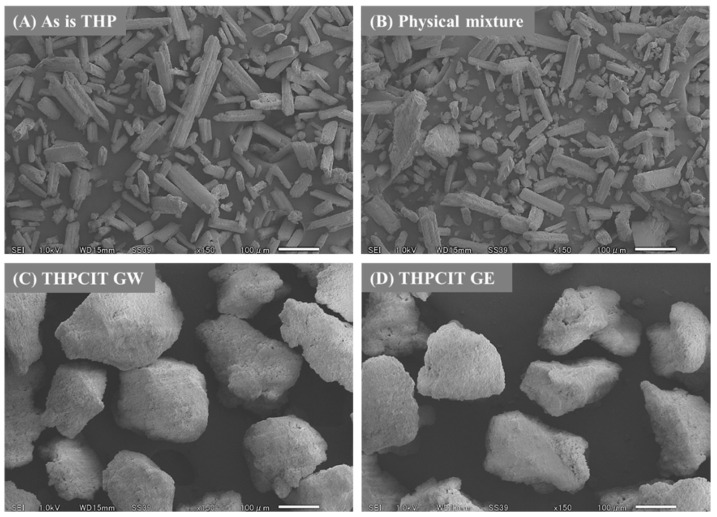
SEM images: (**A**) “As is” THP, (**B**) a physical mixture consisting of THP, CIT, and HPC bulk powder, (**C**) dried RAG granules formed using water (GW), and (**D**) the granules formed using ethanol (GE).

The SEM images and physical properties provided confirmation of the granule formation. “As is” anhydrous THP exhibits a needle-like morphology, and physical mixture composed of the crystals (Figure 4A,B). The RAG provided dense and heavy granules, which were approximately over 200 μm as shown in Figure 4C,D. This finding was supported by the increase in apparent density and decrease in tapped density, as a result of RAG (Table 1). The decrease in angle of repose (AR) and the increase in both the Hausner ratio (HR) and Carr index (CI) also contributed to an improvement of flowability. These values in dried RAG granules indicate “good” or “fair” score as a granule [38,39].

**Figure 5:**
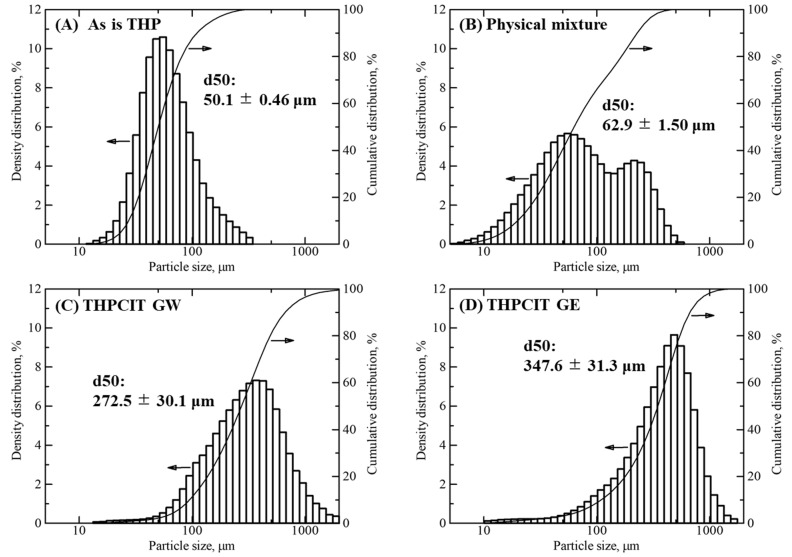
Volume-based particle size distribution (PSDs) and median diameters (d50): (**A**) “As is” THP, (**B**) a physical mixture consisting of THP, CIT, and HPC bulk powder, (**C**) dried THPCIT granules using water (GW), and (**D**) the granules using ethanol (GE) prepared by RAG. The value of d50 is given in the figure (mean ± SD; *n* = 3).

To assess the granule size in detail, the particle size distribution (PSD) of granule was determined. The sample in the dry state was analyzed by laser diffraction (Figure 5). In agreement with the previous results, it was found that the grown granule was obtained by RAG. Interestingly, the PSDs of GW and GE granules resulted in different observations. The median diameter (d50) of GW granules was 272.5 ± 30.1 μm, which was smaller than that of GE granules (347.6 ± 31.3 μm). Additionally, the PSD of GW granules was broader due to the smaller particle sizes (Figure 5C), whereas the GE granules exhibited a steeper distribution curve. Wet GW granules were dehydrated via a drying process during which fine powder was produced [40,41]. On the other hand, GE granules had no dehydration process. Therefore, the dried GW granules included a fraction of finer particles compared to the GE granules.

### 3.4. X-ray CT Micrographs of Tablets

**Figure 6:**
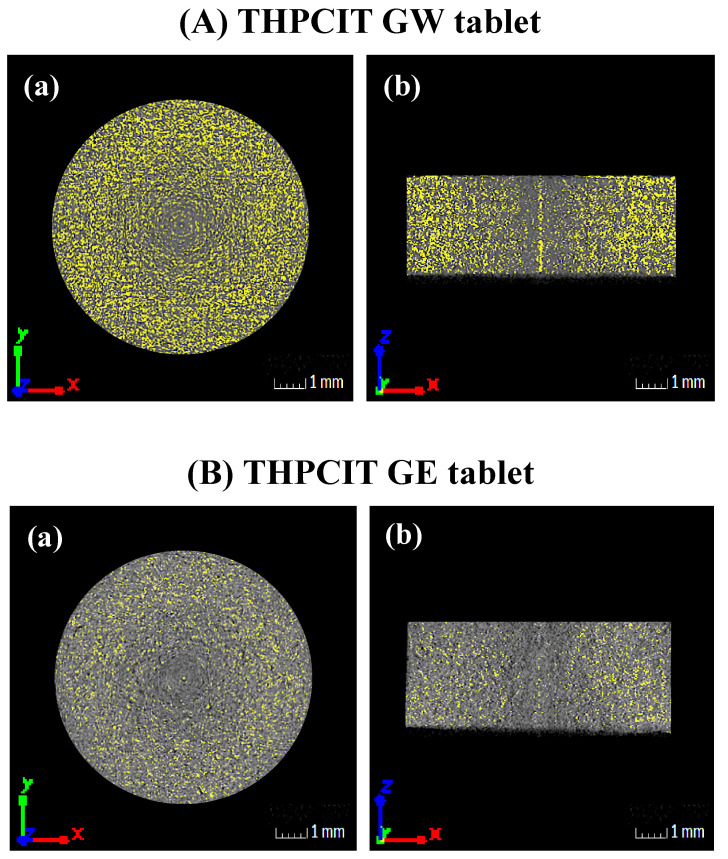
X-ray CT images of (**A**) GW and (**B**) GE tablets at (**a**) transverse and (**b**) longitudinal section. The color gradient from black to gray indicates the gradient from low to high absorption. The higher absorption area, i.e., higher density area, is mapped as yellow.

The image scanned by X-ray CT was used to analyze the internal density in the tablets compressed from RAG granules. As shown in Figure 6A, the GW tablet was mapped as a yellow color, suggesting a high-density characteristic. The image also exhibited a high-density area in the outer portion of the tablet, whereas the center of the tablet was less dense. As explained earlier, the dried GW granule contained a fine particle fraction due to dehydration of the cocrystal hydrate. It has been reported that fine particles contribute to the formation of tablets with higher density [42,43]. In the case of the GE tablet, the image revealed that the density was lower than that of the GW tablet because there was no phase transition (Figure 6B).

### 3.5. Hardness, Disintegration, and Dissolution Ability of Cocrystal Tablets

The hardness and disintegration time of the tablets were influenced by the density, which was measured by X-ray CT as described in an earlier section. The relatively low-density GE tablet exhibited adaptive hardness, while the dense GW tablet had higher hardness, resulting in a drastic delay of the disintegration time during dissolution testing (Table 2). These findings were perfectly in proportion to the findings of the density analysis.

**Figure 7:**
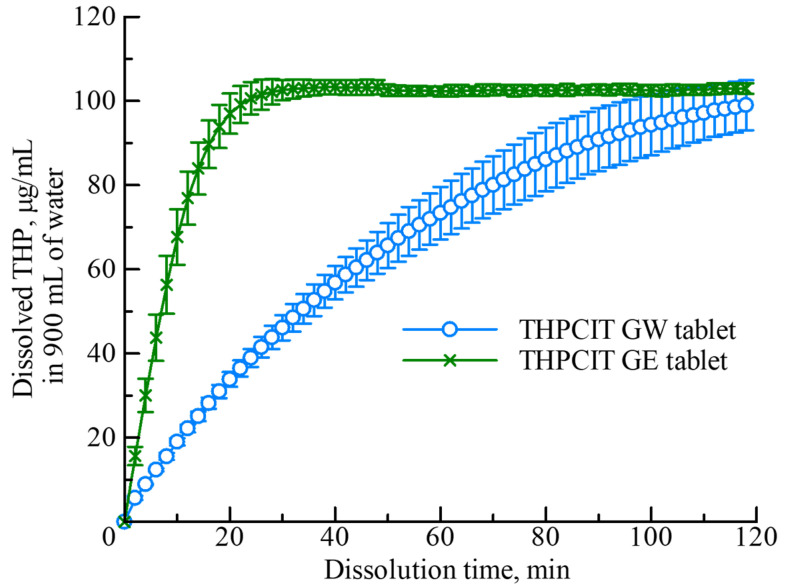
Concentration of THP as a function of the dissolution time of THPCIT cocrystal tablets formed from GW (light blue circles) and GE (dark green crosses) in 900 mL water (mean ± SD; *n* = 3).

The dissolution behavior of the GW and GE tablets in aqueous medium was compared (Figure 7). In both tablets, the disintegrated sample was completely dissolved, and the concentration of THP reached almost 100% (104.44 μg/mL) at the final timepoint (120 min). However, the dissolution of the GW tablet was significantly slower than that of the GE tablet, because of the longer disintegration time (Table 2). The rate-determining step in the disintegration of porous tables is the liquid penetration [44]. Thus, the penetration in the GW tablet was hindered because of the high-density structure.

Additionally, phase transition during the dissolution of the cocrystal can be explained by the reported insight [45,46]. In this study, it was considered that: (i) the anhydrous THPCIT cocrystal was rapidly dissociated to amorphous or nanocrystalline anhydrous THP clusters, after the dissolution of soluble CIT; and (ii) the THP clusters were transformed to monohydrate [45]. The GW tablet contained fine powder of anhydrous cocrystal as explained earlier, thus the specific surface area was larger and the surface of the tablet was easily converted into THP monohydrate during dissolution testing compared to GE tablet. The THP monohydrate layer inhibits the penetration of aqueous medium, leading to disintegration and dissolution delay [20,47]. The amount of penetrated liquid W can be also expressed by the following Lucas–Washburn equation:

(3) $$W^{2}=\frac{\rho_{liq}^{2}A_{ave}^{2}\gamma \cos\theta }{2\eta S}\frac{\varepsilon^{3}}{1-\varepsilon }t$$

where $\rho_{liq}$ and η are the density and viscosity of the liquid, respectively; γ and θ are the surface tension and contact angle between the liquid and particle layer, respectively; $A_{ave}$, S and ε are the averaged sectional area, specific surface of the particle layer, and porosity, respectively; and t is time [48,49,50]. This equation indicates that large specific surface area on the surface of tablet results in low water penetration into tablet. In previous study, the wettability of 1:1 flufenamic acid-nicotinamide cocrystals enhanced through the application of semicrystalline polymers in the matrix, subsequently the significant increase of flufenamic acid dissolution was observed [51]. Therefore, the inhibition of water penetration (wettability) in GW tablet poses a challenge for the dissolution improvement. In regard to the GE tablets, which were prepared from anhydrous cocrystal granules without any phase transition, the specific surface area was smaller and aqueous medium could easily penetrate the tablet compared with the GW. In summary, the GE tablet reached a maximum concentration of THP quickly.

## 4. Conclusions

Complete theophylline–citric acid (THPCIT) cocrystal formation (>99%) and adequately sized granules (d50: >250 μm) were simultaneously realized by the resonant wet acoustic granulation (RAG) system, while the previous method of high-shear wet granulation (HSWG) resulted in a partial reaction between THP and CIT and low cocrystal yield. RAG can accomplish powerful and rapid mixing of materials. THP formed either hydrate or anhydrous cocrystals with CIT, depending on the choice of granulation liquid (water or ethanol). The granulation using water (GW) facilitated the cocrystal hydrate formation, but the resulting crystals were transformed into anhydrous cocrystals after drying. On the other hand, the granulation using ethanol (GE) resulted in the formation of anhydrous cocrystals before and after drying. When the dried GW granules including fine powder due to dehydration were compressed, the hardness and density increased and resulted in slower dissolution than by compression of GE granules. These results demonstrated that the physical properties were changed depending on the granulation liquid used during RAG. Our newly developed RAG system will contribute to the required reduction in the number of operations.

## Figures and Tables

**Table 1:** Effect of granulation on the physical properties (*n* = 3).

	*ρ*_A_^1^, g/cm^3^	*ρ*_T_^2^, g/cm^3^	AR ^3^, °	HR ^4^	CI ^5^, %
As is THP	2.47 ± 0.17	5.06 ± 0.97	58.0 ± 6.00	2.03 ± 0.26	50.3 ± 6.23
Physical mixture	2.82 ± 0.17	5.69 ± 0.21	58.4 ± 6.23	2.02 ± 0.06	50.5 ± 1.42
THPCIT GW	4.23 ± 0.10	4.92 ± 0.19	36.8 ± 4.03	1.17 ± 0.02	14.4 ± 1.57
THPCIT GE	4.52 ± 0.39	4.93 ± 0.40	31.9 ± 3.89	1.09 ± 0.01	8.27 ± 0.92

^1^ Apparent density, ^2^ tapped density, ^3^ angle of repose, ^4^ Hausner ratio, ^5^ Carr index.

**Table 2:** Hardness and disintegration time of THPCIT cocrystal tablets formed from GW and GE (*n* = 3).

	Hardness, N	Disintegration Time, min
THPCIT GW tablet	74.0 ± 5.0	41.1 ± 6.5
THPCIT GE tablet	24.7 ± 2.1	8.7 ± 1.0

## Data Availability

The data presented in this study are available on request.
